# Effectiveness and safety of traditional Chinese medicine in the treatment of senile hypotension: A protocol for systematic review and meta-analysis

**DOI:** 10.1097/MD.0000000000025385

**Published:** 2021-04-23

**Authors:** Zhijun Chen, Siyuan Zhu, Meihua Liu, Genhua Tang, Zhiying Zhong, LunBin Lu

**Affiliations:** a The Affiliated Hospital of Jiangxi University of Traditional Chinese Medicine; b Jiangxi University of Traditional Chinese Medicine, Nanchang, China.

**Keywords:** meta-analysis, orthostatic hypotension, protocol, senile hypotension, systematic review, traditional Chinese medicine

## Abstract

**Background:**

Senile hypotension refers to those whose blood pressure (BP) is lower than 90/60 mm Hg. The consequences can not only cause insufficient cardio-cerebral perfusion, but severe cases may also cause angina pectoris and stroke. At present, Western medicine has no ideal therapeutic drugs for senile hypotension. The aim of this systematic review is to assess the effectiveness and safety of traditional Chinese medicine (TCM) therapy for senile hypotension.

**Methods:**

Two reviewers will electronically search the following databases: the Cochrane Central Register of Controlled Trials; PubMed; EMBASE; China National Knowledge Infrastructure; Chinese Biomedical Literature Database; Chinese Scientific Journal Database (VIP database); and Wan-Fang Database from the inception, without restriction of publication status and languages. Additional searching including researches in progress, the reference lists and the citation lists of identified publications. Study selection, data extraction, and assessment of study quality will be performed independently by 2 reviewers. If it is appropriate for a meta-analysis, RevMan 5.4 statistical software will be used; otherwise, a descriptive analysis will be conducted. Data will be synthesized by either the fixed-effects or random-effects model according to a heterogeneity test. The results will be presented as risk ratio (RR) with 95% confidence intervals (CIs) for dichotomous data and weight mean difference (WMD) or standard mean difference 95% CIs for continuous data.

**Results:**

This study will provide a comprehensive review of the available evidence for the treatment of TCM with senile hypotension.

**Conclusions:**

The conclusions of our study will provide an evidence to judge whether TCM is an effective and safe intervention for patients with senile hypotension.

**Ethics and dissemination:**

This systematic review will be disseminated in a peer-reviewed journal or presented at relevant conferences. It is not necessary for a formal ethical approval because the data are not individualized.

**Trial registration number:**

INPLASY2020110091.

## 1 Introduction

### 1.1 Description of the condition

Senile hypotension refers to cardiovascular diseases with systolic blood pressure (BP) <90 mm Hg and (or) diastolic BP<60 mm Hg as the main clinical manifestations that occur in the elderly, and it is a common type of hypotension.^[1]^ It can be accompanied by obvious dizziness, nausea, fatigue, drowsiness, chest tightness and other syndromes, accounting for about 15% to 20% of the elderly, and it is more common in clinical practice. According to statistics, senile hypotension accounts for about 10% to 20% of elderly people over 65 years old, and orthostatic hypotension (OH) is more common.^[2–4]^ Some foreign studies have shown that the mortality rate of the elderly has a “J” shape relationship with BP, and the mortality rate of people with hypotension is higher than that of people with normal BP.^[5]^ Senile hypotension can not only cause insufficiency of cardio-cerebral perfusion, but also can cause angina pectoris and stroke in severe cases.^[6]^ For patients with chronic hypotension, Western medicine has no ideal therapeutic drugs.^[1]^ There is no name for hypotension in Chinese medicine. According to its typical clinical manifestations, Chinese medicine categorizes it into categories such as “vertigo” and “fatigue”. At the same time, Chinese medicine believes that it belongs to the deficiency of both qi and yin, weak kidney qi, insufficient kidney yang, and lack of heart qi causes dizziness, fatigue, and palpitations after exercise. And the deficiency of kidney yin cannot nourish the organs.^[7]^ Because the elderly population tends to be weak, senile hypotension is more common, and aging is getting worse, so how to treat senile hypotension and improve the health of the elderly is of great value.^[4–5]^ The regulation of BP by traditional Chinese medicine (TCM) is a commonly used treatment method, which is widely accepted and recognized worldwide. According to long-term clinical observations, the treatment of senile hypotension with warming of the heart, spleen and kidney yang is effective.^[8]^

### 1.2 Description of the intervention

Hypotension is mainly manifested as shortness of breath, fatigue, weak pulse, etc. The treatment is mostly based on replenishing qi and blood. TCM can promote the body to achieve a relative balance through overall adjustment and dialectical treatment to achieve the purpose of curing diseases. Such as: Shenmai injection. It is a modern Chinese medicine preparation made from the ancient prescription “Shengmaiyin”. It is mainly extracted and refined from Chinese medicinal materials such as red ginseng and Ophiopogon japonicus. According to the theory of TCM, it has the functions of replenishing qi and solidifying, nourishing yin and promoting fluid, nourishing the heart and rejuvenating the pulse.^[9]^ At the same time, modern pharmacological studies have found that it has the effects of enhancing myocardial contractility to increase cardiac output, dilating coronary arteries to increase myocardial blood supply, improving microcirculation, and increasing blood oxygen saturation. Therefore, it is widely used in the treatment of heart failure, arrhythmia and other cardiovascular diseases.^[10–13]^ On this basis, it can be considered that Chinese herbal medicines such as Shenmai injection are ideal drugs for the treatment of elderly hypotension.^[14]^ Therefore, TCM may become an effective treatment to delay the progression of senile hypotension.

### 1.3 How the intervention might work

TCM treatment of senile hypotension mainly focuses on warming and invigorating heart yang, kidney yang, and strengthening spleen yang. Qi is the motive force for blood movement, qi is yang, and strong heart yang results in greater ejection power from the heart, which is the guarantee for raising BP. Deficiency of spleen yang results in clusters of damp pathogens, damp pathogens trapping the spleen without qi and blood, and the blood vessels are not filled and the force on the blood vessel wall is reduced. Deficiency of kidney yang leads to decreased visceral function, qi and blood failure leads to empty pulse channels, and weak heart yang leads to weak motivation. Weak pulse will lead to hypotension. Deficiency of the heart and spleen is the standard, and deficiency of the kidney yang is the basis.^[8]^

### 1.4 Why it is important to do this review

Patients with orthostatic hypotension (OH) are mainly frail and sickly elderly people who take other large amounts of drugs at the same time. However, by far the most common comorbidity in patients with orthostatic hypotension is hypertension, and about 70% of elderly patients with hypotension suffer from hypertension.^[15]^ Conversely, OH is present in about 10% of patients referred to hypertension specialists.^[16]^ In community studies, hypertension is closely related to OH.^[17]^ The coexistence of hypertension and orthostatic hypotension makes both diseases difficult to manage, because treatment of 1 disease may worsen the condition of the other.^[18]^ However, the advantage of TCM intervention lies in the overall treatment, which stimulates the body's own defense capabilities and makes the body's own balance.

### 1.5 Objectives

To systematically evaluate the effectiveness and safety of TCM therapy for senile hypotension patients.

## 2 Methods and analysis

This protocol was designed in accordance with the methodological guidelines for overviews provided by the Cochrane Handbook for Systematic Reviews of Interventions.^[19]^ It is registered on the International Prospective Register of Systematic Reviews. (Registration number INPLASY2020110091; https://inplasy.com/inplasy-2020-11-0091/.)

### 2.1 Inclusion criteria for study selection

#### 2.1.1 Types of studies

Randomized controlled trials will be included, without restrictions on publication status.

#### 2.1.2 Types of participants

Adult patients with senile hypotension, regardless of sex, race, or educational and economic status.

#### 2.1.3 Types of interventions

Research using TCM therapy, without limiting the treatment time and dose.

#### 2.1.4 Types of comparisons

The control group's treatment is not limited, including no treatment, placebo, or any control considered for comparison in a single systematic review.

#### 2.1.5 Types of outcome measures

Primary outcomes: Before and after BP

Secondary outcomes:

1. Heart rate
2. Cardiac output

## 3 Clinical efficacy

### 3.1 Search methods for identification of studies

#### 3.1.1 Electronic searches

The following databases from the inception to December 2020 will be searched by 2 independent reviewers, without restriction to publication status and languages: the Cochrane Central Register of Controlled Trials; PubMed; EMBASE; China National Knowledge Infrastructure; Chinese Biomedical Literature Database; Chinese Scientific Journal Database (VIP database); and Wan-Fang Database. A search strategy for PubMed database, which is established according to the Cochrane handbook guidelines, is shown in Table 1. Similar search strategies will be applied for the other databases. Before this review completed, the 2 reviewers will conduct the searching once again to ensure the latest studies could be included.

**Table 1:** Search strategy for the PubMed database.

Number	Search terms
1	Senile hypotension
2	Elderly hypotension
3	hypotension
4	Orthostatic hypotension
5	blood pressure
6	or 1–5
7	traditional Chinese medicine
8	traditional Chinese medicine Injection
9	or 7–8
10	6 and 9

#### 3.1.2 Searching other resources

Besides, electronic sources for relevant researches in progress will also be searched, including Clinicaltrials.gov (http://www.clinicaltrials.gov) and the World Health Organization International clinical trials registry search portal (http://apps.who.int/trialsearch/). Additionally, the citation list will be retrieved in Web of Science. Besides, the reference lists of those studies meeting the inclusion criteria and relevant systematic reviews will also be identified for additional relevant studies.

### 3.2 Data collection and analysis

#### 3.2.1 Selection of studies

**Figure 1:**
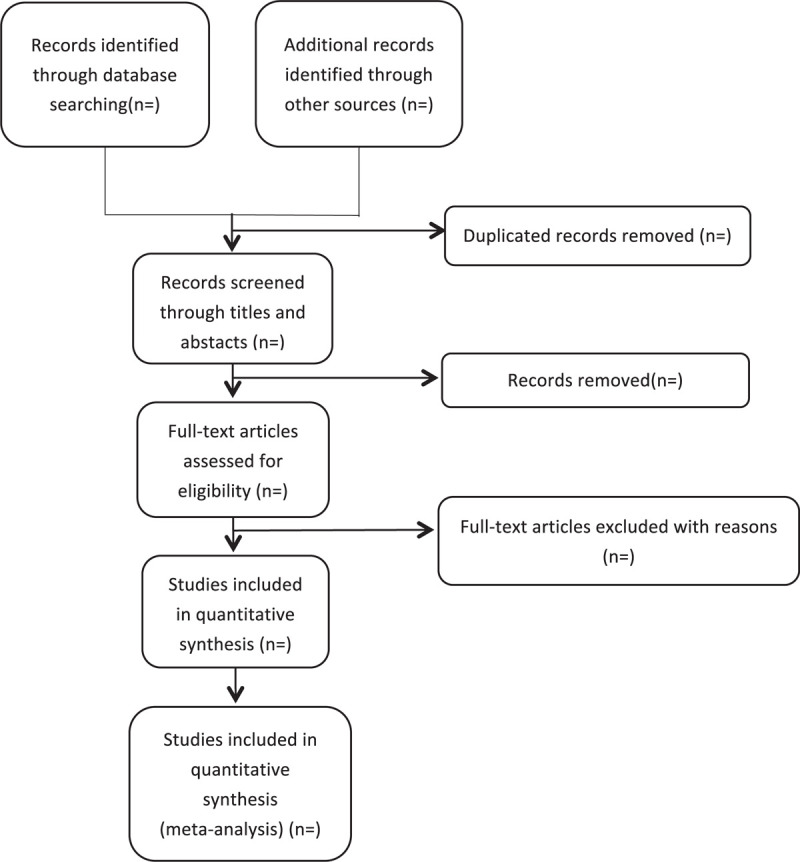
Flowchart of literature selection.

We plan to conduct this systematic review between December 30, 2020 and July 30, 2022. All reviewers have undergone a training to ensure a basic understanding of the background and purpose of the review. After electronic searching, the records will be uploaded to a database set up by EndNote software (V.X7). Records selected from other sources will also be moved to the same database. Two reviewers (SYZ and GHT) will independently screen the titles, abstracts, and keywords of all retrieved studies and decide which trials meet the inclusion criteria. We will obtain the full text of all possibly relevant studies for further assessment. Excluded studies will be recorded with explanations. Any disagreements will be resolved by discussion between the 2 reviewers (SYZ and GHT) and the third author (ZJC) for arbitration when necessary. We will contact reviewers of trials for clarification when necessary. The study flow diagram is shown in Figure 1.

### 3.3 Data extraction and management

A unified data extraction form will be designed by all of the reviewers and its applicability will be tested in a small scope of trials by 2 reviewers (ZYZ and LBL). They will then independently extract data in the following domains: general information, participants, methods, interventions, outcomes, results, and other information. Any disagreement will be discussed between the 2 reviewers, and further disagreements will be arbitrated by the third author (ZJC).

### 3.4 Assessment of risk of bias in included studies

The risk of bias will be assessed by 2 reviewers (ZJC and MHL) with the Cochrane Collaboration's tool for risk of bias assessment. The risk of bias in included studies will be evaluated according to the following aspects: sequence generation, allocation sequence concealment, blinding of participants and personnel and outcome assessors, incomplete outcome data, selective outcome reporting, and other sources of bias. Theassessments will be classified into 3 levels: low risk, high risk, and unclear risk.

### 3.5 Measures of treatment effect

RevMan V.5.4 will be used for data analysis and quantitative data synthesis. For continuous data, we will use standard mean difference to measure the treatment effect with 95% confidence intervals (CIs). For dichotomous data, a risk ratio (RR) with 95% CIs for analysis will be adopted.

### 3.6 Unit of analysis issues

Data from studies with parallel-group will be included for meta-analysis. For randomized cross-over trials, only the first phase data will be included. In these trials, participants are individually randomized to 2 intervention groups, and a single measurement of each outcome from each participant is collected and analyzed.

### 3.7 Dealing with missing data

We will try to contact the first or corresponding authors of the included studies by telephone or email to retrieve missing or insufficient trial data. If missing data are unavailable, we will make an assumption using the terms “missing at random” and “not missing at random” to represent different scenarios, which is recommended in the Cochrane Handbook.^[20]^ For the data “missing at random,” only the available data will be analyzed. For the data “not missing at random,” we will displace the missing data with replacement values and a sensitivity analysis will be used to determine whether the results are inconsistent.

### 3.8 Assessment of heterogeneity

On the basis of the data analysis, random effect or fixed effect models will be employed according to the heterogeneity given by *I*^*2*^ statistic value. To be concrete, a fixed effect model will be adopted if the heterogeneity is indicated as high (*I*^*2*^ < 50%); otherwise, a random effect model will be applied on the contrary.

### 3.9 Assessment of reporting biases

We will use funnel plots to detect reporting biases and small study effects. If more than 10 studies are included in the meta-analysis, we will conduct a test for funnel plot asymmetry using Egger method. All eligible trials will be included, regardless of their methodological quality.

### 3.10 Data synthesis

The systematic review will be conducted with the use of RevMan 5.4. Taking account of the heterogeneity assessment, MD or RR with fixed or random effect model will be computed. Additionally, if heterogeneity is considered significant, the sensitivity or subgroup analysis will be generated to distinguish the source of it. When it comes to the situation that the data are insufficient for quantitative analysis, the review will only represent and summarize the evidence.

### 3.11 Sensitivity analysis

Sensitivity analysis will be conducted to validate the robustness of the primary results. We will exclude certain trials by a reevaluation of methodological quality, study types, sample size, missing data or other possible factors. Careful interpretations will be employed for sensitivity analysis if differ substantially.

### 3.12 Grading the quality of evidence

The Grading of Recommendations Assessment, Development and Evaluation (GRADE) working group methodology will be applied for the quality of evidence for all outcomes.^[21]^ Six domains will be assessed, containing risk of bias, consistency, directness, precision, publication bias and additional points. The assessments will be categorized into 4 levels: high, moderate, low, or very low.

### 3.13 Subgroup analysis

If data are available, a subgroup analysis will be performed based on the type of moxibustion intervention (TCM injection, Chinese medicine decoction, Chinese medicine granules, etc) because this is the main factor causing heterogeneity.

## 4 Discussion

Senile hypotension is mostly due to old age, physical weakness, low activity, low metabolism, and secondary to senile diseases. The main clinical manifestations are dizziness, fatigue, sore limbs, palpitations after exercise, chest tightness, shortness of breath, etc.^[22]^ As the immune system of elderly patients declines, and most of them suffer from multiple diseases at the same time, Western medicine is often not effective in treating senile hypotension. However, the treatment of TCM is mainly to improve one's own righteousness, which can promote the body to a relatively balanced state through the overall adjustment method, which has a certain effect. With the continuous advancement of medical treatment and the acceleration of aging, cardiovascular diseases, which are dominated by the elderly, have received unprecedented attention. Especially with the strengthening of the awareness of the risk of cerebrovascular accidents caused by hypotension in recent years, the treatment of senile hypotension has also become a vital part.^[9]^ Therefore, it is of great significance to explore the effectiveness and safety of TCM in treating senile hypotension.

## Author contributions

**Conceptualization:** Zhijun Chen, Siyuan Zhu.

**Data curation:** Zhijun Chen, Siyuan Zhu, Meihua Liu, Genhua Tang, Zhiying Zhong, Lunbin Lu.

**Formal analysis:** Siyuan Zhu, Meihua Liu.

**Investigation:** Genhua Tang, Zhiying Zhong.

**Methodology:** Zhijun Chen, Siyuan Zhu, Lunbin Lu.

**Software:** Siyuan Zhu, Meihua Liu.

**Supervision:** Zhijun Chen, Meihua Liu, Lunbin Lu.

**Writing – original draft:** Zhijun Chen, Siyuan Zhu.

**Writing – review & editing:** Zhijun Chen, Meihua Liu, Genhua Tang, Lunbin Lu.
